# Increased cardiovascular risk factors in breast cancer survivors identified by routine measurements of body composition, resting heart rate and arterial blood pressure

**DOI:** 10.1186/2193-1801-3-150

**Published:** 2014-03-19

**Authors:** David H Jones, Melisa Nestore, Sara Henophy, Julia Cousin, Alain Steve Comtois

**Affiliations:** Department of Biological Sciences, University of Quebec in Montreal, Montreal, QC H3C 3P8 Canada; Department of Exercise Science, The Richard J. Renaud Science Complex, Concordia University, Montreal, QC H4B 1R6 Canada; Integrative Health & Wellness, VM Medical Centre, Montreal, QC H3G 1L5 Canada; Department of Kinanthropology, University of Quebec in Montreal, Montreal, QC H3C 3P8 Canada

**Keywords:** Breast cancer, Waist circumference, Blood pressure

## Abstract

**Purpose:**

The main objective of this prospective study was to obtain a better understanding of the body composition and vital sign measures of cancers survivors (CS) when compared to regular (R) patients.

**Methods:**

A total of 9,315 female patients were evaluated: 476 CS and 8,839 R patients. Kinesiologists worked side by side with the medical/oncology team to collect a number of base-line measurements on body composition, resting heart rate, and blood pressure as part of the standard intake evaluation during the female patients’ annual checkup.

**Results:**

CS were more likely to have a higher BMI (P = 0.001) and a larger waist circumference (P = 0.001) than R patients. CS were also shown to have higher blood pressure values: diastolic pressure of 76.9 mmHg ± 10.5 VS 75.5 mmHg ± 9.9, (P = 0.01) and systolic pressure of 129.8 mmHg ± 17.2 VS 126.7 mmHg ±17.4 (P = 0.001) compared to R patients, respectively. Regression analysis looking at the relationship between mean arterial pressure and waist circumference did not show any difference between the two groups (CS vs R).

**Conclusion:**

CS who had a higher BMI, a larger waist circumference and higher blood pressure levels, are probably at greater risk for developing cardiovascular disease, diabetes, various musculoskeletal problems as well as an increased risk for various forms of cancers including reoccurrence of previously treated cancer when compared to R patients. Changes in body composition should be considered by the medical team when looking at preventative healthcare strategies for their CS patients.

## Introduction

It has been well established that as body composition changes with an increase in waist circumference and an overall increase in body mass index (BMI), there is a greater risk for individuals to develop various metabolic diseases such as diabetes, heart disease, as well as an increased risk of developing cancer or having a reoccurrence of a previously treated cancer (Han et al. 2006). Major health organizations, such as the World Health Organization (WHO), have recognized the importance of physical activity, maintaining ideal body size, improving dietary habits and ceasing smoking in order to reduce the risk of developing various metabolic diseases (Harvie et al. 2005). Unfortunately even with these guidelines, many people still present with increases in total body fat, increases in BMI, as well as reduced fitness levels (Flegal et al. 2010).

Numerous studies have looked at the relationship between changes in body composition and vital sign measures and the increase in risk for developing various diseases. Most of these studies, however, have had to rely on patients self-reporting their values or have had to rely on data extraction from records kept at multiple locations that may tend to underestimate the patient’s body composition values such as body weight and body fat (Battaglini et al. 2011; Blair et al. 2012). Other investigators have used meta-analysis to evaluate the impact of risk and body composition (Carmichael and Bates 2004). Thus, we hypothesized that by measuring body composition and vital signs in women as part of their regular follow-up care and comparing the Cancer Survivors (CS) to the Regular Patients (R) that we would be able to obtain a better understanding of the potential increase of ill health risk that cancer patients face after completing therapy. The main objective of this prospective study was to obtain a better understanding of the body composition and vital sign measures of CS when compared to R.

## Methodology

All participants were recruited at the VM medical centre (VMMC). As a patient in the VMMC system, patients were encouraged to read over and consider signing a consent form so that data collected by the medical team could be analyzed for trends. All of the data that was collected and passed over to the research team was anonymous. The consent form was approved for usage by the VMMC ethics committee and conforms to the Helsinki declaration on research on human subjects.

A protocol was developed in-house that encouraged patients, waiting for their annual medical appointments, to undergo body composition and blood pressure measurements that once collected would be entered into the centre’s electronic charting system. All of the tests were administered by licensed and experienced kinesiologists who were comfortable working with special populations. The information collected on each patient was made accessible to their physician so that they may have a better understanding of some of the patient’s health challenges.

A testing room was set up close to the patient waiting area at the VMMC. Patients were brought to the testing room and given an over view of the type of tests that would be performed. This period of time allowed the patients to relax and ask any questions before testing began.

An automated blood pressure cuff (Physiologic Auto-memory 90, Montreal, Canada) was used to measure resting heart rate and blood pressure (BP). Most patients were tested on their left arm, unless they were oncology patients who had undergone breast surgery along with lymph node removal on their left side. These patients were tested using their right arm. All BP measurements were performed with the forearm supported at heart level with an appropriate size cuff wrapped around the patient’s upper arm. The cuff was aligned with the brachial artery. The kinesiologists followed the Canadian guidelines when applying and recording measurements. If the measurements were considered outside of the expected values for that patient (based on their age) the patient would relax for 5 minutes after which the test was repeated. This is consistent with the Canadian Heart and Stroke Foundation Guidelines (Hypertension Canada 2013).

The patient was then asked to stand in front of a stadiomometer (Health O Meter, McCook, IL, USA) for measuring height. Briefly, it consisted of a vertical ruler with a sliding horizontal rod that was adjusted so that it would rest on the top of the head while the participant would take in a full breath and hold for several seconds while the measured height was recorded. Total body weight was recorded with a floor scale (Am Cells, Rice Lake weight scales, Rice Lake Wisconsin, USA).

The waist was measured following ACSM guidelines (American College of Sports Medicine 2009). Waist circumference was measured using a Gulick tape (Heart and Stroke Foundation, Ottawa, ON, Canada). Waist measurement was performed with the patient standing upright, feet together, and arms at their side while maintaining a relaxed breathing pattern. A horizontal measure was taken at the narrowest location between the umbilicus and the sternum. The test was repeated two times and the average was taken. If there was a difference of more than 5 millimeters between measurements, a third measurement was taken.

For this study patients were classified as either being CS or R. All of the women classified as CS underwent either a partial or a full mastectomy, which possibly included additional forms of adjuvant therapy. The additional therapies may have included chemotherapy, radiotherapy and hormonal therapy. Some patients may have had only one additional therapy while other patients may have had two or three different forms of adjuvant therapy. Patients classified as R would not have had any of the adjuvant therapies listed above. Both the CS and the R may also have had other health issues and were taking additional medication that may have affected outcome measurements.

### Data analysis

Statistical analysis was performed using IBM SPSS Statistics (Version 19.0). Simple unpaired t-tests were performed on dependent variables between groups (CS vs R). Pearson correlation was used to evaluate relationships between outcomes measures. Regression analysis was performed using Sigma Plot for Windows (Version 11.0). Statistical significance was set at p < .05.

## Results

A total of 9,315 female patients were evaluated: 476 CS patients and 8,839 R patients. The mean age of the patients was significantly different being CS = 59.71 ± 9.93 yrs. and R = 55.81 ± 9.90 yrs. (p = 0.00).

A total 338 CS patients had a partial mastectomy, 62 CS patients had a full mastectomy, 54 CS patients had reconstructive surgery and for 22 CS patients, the surgical intervention was unclear. The right side was the surgically affected side for 213 CS patients, left side was the surgically affected side for 239 CS patients, 19 CS patients had both sides affected and for 5 of the CS patients the affected side was unclear.

Surgery was the only intervention for 35 CS patients, 7 CS patients had surgery along with chemotherapy, 32 CS patients had surgery along with radiotherapy, 49 CS patients had surgery along with hormone therapy and 345 CS patients had surgery along with multiple forms of therapy, which included combinations of chemotherapy, radiotherapy and hormonal therapy. For 9 CS patients the treatment was not clearly indicated.

Significant differences between CS and R in the following outcome measures are shown in Table 1, where Diastolic pressure: CS = 76.9 ± 10.53 VS R = 75.5 ± 9.91 mmHg (p = 0.014), Systolic pressure: CS = 129.8 ± 17.18 VS R = 126.7 ± 17.42 mmHg (p = 0.001). Mean Arterial Pressure CS = 93.72 ± 14.27 R = 92.14 ± 12.85 (p = 0.001). BMI: CS = 26.99 ± 5.14 VS R = 25.97 kg/m^2^ ± 5.61 (p = 0.000) and waist circumference CS = 88.44 ± 11.83 VS R = 86.29 ± 11.98 cm (P = 0.000). Resting heart rate was not significantly different between the two groups: CS = 73.25 ± 12.82 VS R = 73.32 ± 11.91 bpm (p = 0.112).

**Table 1:** **Anthropometric and vital signs comparison between all regular patients and all cancer survivors**

	R n = 8839	CS n = 476	p
Diastolic BP (mmHg)	75.52 (9.91)	76.85* (10.53)	0.01
Systolic BP (mmHg)	126.70 (17.42)	129.83* (17.18)	0.00
Mean arterial pressure (mmHg)	92.14 (12.85)	93.72* (14.27)	0.01
Resting heart rate (bpm)	72.32 (11.91)	73.85 (12.82)	0.10
Weight (kg)	67.49 (13.71)	68.97* (13.54)	0.02
Height (cm)	161.18 (6.76)	159.64* (6.41)	0.00
BMI	25.97 (5.61)	26.99* (5.14)	0.00
Waist circumference (cm)	86.29 (11.98)	88.44* (11.83)	0.00

Mean ± (SD), *significantly different to R (indicated in p column), CS = cancer survivors, R = regular patients.

As shown in Tables 2 and 3, patients were stratified according to 10-year age segments from 20–29 years to 80–89 years. At each end of the stratification there were only a few patients in the CS group: 1 patient in the 20–29 year, 7 patients in the 30–39 year range and 5 patients in the 80–89 year range. According to age groups, significant differences were seen in the diastolic blood pressure for the 40–49 and the 50–59 age groups (p = 0.00 and p = 0.01, respectively), as well as significant differences for weight, BMI and waist circumference in the 50–59 age range (p = 0.00, p = 0.03 and p = 0.05, respectively). In the 80–89 age group, the CS group diastolic and systolic blood pressure and the waist circumference were all significantly less (p = 0.01, p = 0.02 and p = 0.04, respectively) in comparison to the R patients.

**Table 2:** **Anthropometric and vital signs measures stratified by 10 yr. age groups (20 to 59 yrs.) for Regular (R) and Cancer Survivors (CS) patients**

Outcome measures	R 20–29 n = 10	CS 20–29 n = 1	R 30–39 n = 255	CS 30–39 n = 7	R 40–49 n = 2311	CS 40–49 n = 78	R 50–59 n = 3224	CS 50–59 n = 140
Mean weight (kg) SD	57.69 (11.32)	57.00 (0.00)	65.14 (13.25)	67.08 (7.26)	67.00 (14.30)	67.23 (14.22)	67.73 (13.86)	70.91* (14.87)
Mean BP diastolic (mmHg) SD	69.70 (5.68)	76.00 (0.00)	71.92 (8.80)	78.14 (9.75)	74.68 (9.89)	78.17* (10.76)	76.14 (9.79)	78.27* (9.92)
Mean BP systolic (mmHg) SD	113.00 (10.51)	129.00 (0.00)	115.65 (11.48)	119.14 (7.86)	119.94 (14.72)	121.33 (16.51)	125.36 (15.77)	127.57 (15.07)
Mean arterial pressure (mmHg) SD	76.48 (26.15)	93.76 (0.00)	86.50 (8.94)	91.81 (8.94)	89.34 (12.32)	92.56* (11.76)	92.68 (12.61)	93.35 (15.43)
Mean heart rate (BPM) SD	78.50 (12.64)	79.00 (0.00)	73.46 (11.34)	71.86 (12.08)	72.22 (11.83)	72.37 (13.63)	72.17 (11.96)	74.06 (12.29)
Mean BMI SD	21.28 (3.47)	21.20 (0.00)	24.28 (5.28)	25.79 (3.68)	25.19 (5.52)	25.48 (5.32)	25.96 (5.60)	27.01 (5.57)
Waist circumference (cm) SD	74.40 (6.07)	76.00 (0.00)	81.82 (10.42)	85.39 (6.96)	83.79 (11.79)	86.02 (11.55)	86.12 (11.85)	88.14* (7.48)

Significance set P < 0.05, Standard deviation = SD, *Significant difference between CS and R with CS being significantly greater than R values.

**Table 3:** **Anthropometric and vital signs measures stratified by 10 yr. age groups (60 to 89 yrs.) for Regular (R) and Cancer Survivors (CS) patients**

Outcome measure	R 60–69 n = 2184	CS 60–69 n = 162	R 70–79 n = 753	CS 70–79 n = 82	R 80–89 n = 102	CS 80–89 n = 5
Mean weight (kg) SD	68.36 (13.16)	68.83 (12.48)	66.97 (12.83)	68.29 (11.98)	62.71 (12.24)	63.44 (6.81)
Mean BP diastolic (mmHg) SD	76.23 (9.79)	76.24 (10.95)	74.85 (10.09)	75.16 (9.90)	74.42 (10.58)	62.60** (9.84)
Mean BP systolic (mmHg) SD	132.41 (18.36)	133.54 (17.16)	138.24 (17.67)	135.56 (17.95)	143.73 (16.43)	126.00** (16.93)
Mean arterial pressure (mmHg) SD	94.53 (13.15)	94.76 (14.11)	95.47 (13.05)	94.14 (15.24)	97.52 (10.75)	83.73** (11.76)
Mean heart rate (BPM) SD	72.79 (11.88)	73.21 (13.65)	71.42 (12.07)	72.74 (11.50)	72.37 (12.15)	74.80 (13.55)
Mean BMI SD	26.66 (5.37)	27.46 (4.90)	26.95 (5.99)	27.81 (4.81)	26.64 (7.12)	24.48 (2.13)
Waist circumference (cm) SD	88.47 (11.70)	89.39 (12.85)	89.53 (12.27)	90.00 (9.53)	90.41 (11.72)	84.86** (4.25)

Significance set P < 0.05, Standard deviation = SD, **Significant difference between CS and R with CS being significantly less than R values.

In general, all CS showed an apparent trend for a higher body weight, BMI and waist circumference when compared to the R patients.

**Figure 1:**
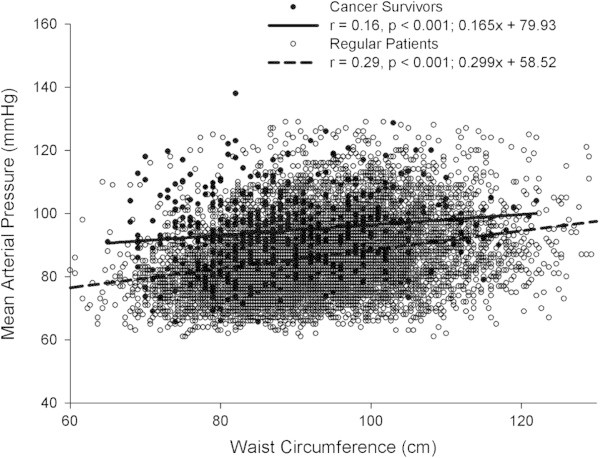
**Relationship between mean arterial pressure and waist circumference in Regular (R) and Cancer Survivor (CS) patients.**

Regression analysis using mean arterial pressure and waist circumference was performed (Figure 1). Significant correlations in the CS and R groups were observed between MAP values and waist circumference values (r = 0.16, p = .001 and r = 0.29, p = .001, respectively).

## Discussion

The CS patients in most of the variables measured were shown to be significantly different from the R patients, with the CS having potentially higher levels of total body fat, a higher BMI and greater waist circumferences. The CS diastolic and systolic blood pressure values were also significantly greater in comparison to the R patients.

Cancer survivors face many challenges as they undergo adjuvant therapy as part of cancer treatment strategies (Yerushalmi et al. 2009). Some studies have shown that treatment has limited to no effect on blood pressure (Stergiou et al. 2002), while other studies have shown that adjuvant therapy may cause significant changes in body composition and elevated blood pressure (Emaus et al. 2010; Thomson et al. 2009). In fact, in the present study, systolic blood pressure for all CS, except for the 30-39 age group (119.14 ± 7.86 mmHg, Table 2) would be classified as pre-hypertensive with values over 120 mm Hg according to ACSM guidelines (American College of Sports Medicine 2013).

The elevated blood pressure may have been related to the adjuvant therapy received by the women. Hormone therapy has been shown to elevate blood pressure in women (Braddock et al. 2013). It is highly likely that some of the patients in the CS group did take hormone replacement medication as part of their adjuvant therapy. Our results on blood pressure are similar to those recently reported by Braddock et al. ( 2013) that have shown that both medication, including chemotherapy, and other adjuvant therapies (radiotherapy) had an effect on blood pressure (Braddock et al. 2013).

### Waist circumference

It has been shown in peri-menopausal and menopaused women that the level of estrogen in the body decreases (Blume-Peytavi et al. 2012; Feigelson et al. 2006). This drop in estrogen has been shown to be linked with an increase in visceral fat (Feigelson et al. 2006). We were able to see this increase in waist circumference in both the CS and R patients. The R patients’ mean waist circumference increased from 81.8 ± 10.42 cm at 30–40 years of age to 89.5 ± 12.27 cm at 70–79 years of age, while in the CS group the mean waist circumferences increased from 85.4 ± 6.96 cm at 30–39 years of age to 90.00 ± 9.53 cm at 70–79 years of age. Waist circumferences were consistently larger for the CS group compared to the R patients except at age 80–89. When evaluating the patient’s weight in relation to their height and their age many of the patients could be considered overweight based on BMI charts (American College of Sports Medicine 2013).

It is well recognized that limited information may be determined through the use of BMI. BMI is a measurement that many physicians use to evaluate a patient’s body composition (Ahn et al. 2012). Therefore it is important to link BMI with additional measurements such as waist circumference (American College of Sports Medicine 2013) to help physicians obtain a better understanding of the patient’s health through the use of body composition measurements. The BMI values obtained on the patients in this study showed that many of the women were not falling into the recommended guidelines put forward by the ACSM (American College of Sports Medicine 2009). The ACSM does make recommendations on waist circumference and BMI and stratifies people according to age group (20–29 yrs, etc.). We observed with both the R and CS patients that their values exceeded the recommended guidelines. As well, there was a small effect of waist circumference increase on mean arterial pressure in CS patients (see Figure 1). In contrast, the increase in waist circumference in R patients appears to have a markedly greater effect on mean arterial pressure. This would also mean that in the lower waist circumference range the mean arterial pressure difference between CS and R patients was larger with the difference becoming smaller as waist circumference increased. A possible explanation for this difference may be associated with treatment the patient underwent (Braddock et al. 2013). It has been shown that various medication types on oncology patients increases the amount of cardiovascular stress on the body (Stergiou et al. 2002). The differences seen in blood pressure and resting heart rate, however, may have been present before treatment.

### Strength of study

Having all women tested by the same team members utilizing the same protocol gives significant strength to this study. The approach used herein contrasts two commonly used approaches, the first being patient self-reporting and the second the use of multiple test sites with multiple teams. From the literature (Dahl and Reynolds 2013), it is known that patients tend to underestimate their weight. The use of patient’s self –reporting data, in particular their own body weight would have increased the amount of error in the data that was collected.

A large number of patients participated in this project, just over 9300 women. This is equivalent to other studies using multiple testers and multiple locations (Hansen et al. 1997). The more locations and the more testers involved in the testing process the greater the risk of error. With three kinesiologists performing all of the measurements we are confident we have minimized some of the intra and inter tester error that could have occurred.

It would be interesting to see the effects of an intervention, such as counseling, while tracking these two groups over time to see if the patients’ body composition and blood pressure would improve. Without any intervention, it would be expected that both body composition and blood pressure values would continue to move in a negative direction as both the CS and R patients aged, with the CS potentially being more affected.

### Limitations of the study

One factor that may have affected vital sign measurements could have been the stress of going to the physician's office for the CS. We speculated that it may have been more stressful for the CS because they were seeing their surgical oncologist as part of an ongoing follow up. The CS patient’s fear of hearing about a possible reoccurrence may have elevated their BP values. On the other hand, the R patients were typically visiting the clinic for an appointment with their GP or their OBGYN as part of their routine yearly evaluation. A second limitation of this study is the homogeneity of the population. There was limited diversity in the population that was tested. Even though it was not specifically recorded most of the women tested were Caucasian and only a small portion of the patients at the VMMC were from another ethnic group. However, the homogeneity of the study population herein may also be considered strength. We recognize that it would have been helpful to have additional information on the patients such as the type of medication that the patients may be ingesting. This confounding variable may influence the outcome measures that were collected on patients in our study. We also understand some of the challenges with collecting this information, such as the level of compliance by the patients in taking medication (Thunander Sundbom and Bingefors 2012), which has been found to be affected by gender and socioeconomic status. Some association may be drawn from the stratification of the patients by age with the expectation that a number of women in both groups will encounter similar health related issue that are associated with age, such as increased risk of osteoporosis (Siris et al. 2014).

### Future directions

Understanding body composition and fitness level has been shown to be strong markers at identifying cancer risk (Kruk and Aboul-Enein 2006). Future studies should incorporate the use of measurement tools, such as impedance units, fat calipers or DEXA for body composition assessment to provide more detailed information about body fat and lean muscle mass. Detailed body composition give a better understanding of body composition of cancer survivors. Nonetheless, using simple vital signs measurements, taken by kinesiologists appears to be sensitive enough to identify individuals at risk. Being able to incorporate the fitness level and activity level of the cancer survivors might also improve sensitivity and may prove important.

## Conclusion

The baseline risk assessment protocol established at the VMMC and administered by kinesiologists is critical in helping to understand some of the fundamental risks faced by patients. The protocol used by the VMMC Kinesiologists, who were working in conjunction with the medical team at the centre, gave the patients a clear starting point as to some of behavior changes to be adopted by the patient to help reduce their risk of developing different forms of cancer, cardiovascular issues and metabolic diseases.
